# Location of metastases in cancer of unknown primary are not random and signal familial clustering

**DOI:** 10.1038/srep22891

**Published:** 2016-03-09

**Authors:** Kari Hemminki, Kristina Sundquist, Jan Sundquist, Akseli Hemminki, Jianguang Ji

**Affiliations:** 1 Division of Molecular Genetic Epidemiology, German Cancer Research Center (DKFZ), Heidelberg, Germany; 2 Center for Primary Health Care Research, Lund University, Sweden; 3 Stanford Prevention Research Center, Stanford University School of Medicine, Stanford, CA, USA; 4 Cancer Gene Therapy Group, Department of Pathology and Transplantation Laboratory, Haartman Institute, University of Helsinki, Finland; 5 Helsinki University Hospital Comprehensive Cancer Center, Helsinki, Finland

## Abstract

Cancer of unknown primary (CUP) is a fatal disease diagnosed through metastases. It shows intriguing familial clustering with certain defined primary cancers. Here we examine whether metastatic location in CUP patients is related to primary non-CUP cancers in relatives based on the Swedish Cancer Registry. Standardized incidence ratios (SIRs) were calculated for CUP patients defined by metastatic location depending on cancer in their first degree relatives. SIRs for CUP were high in association with liver (3.94), ovarian (3.41), lung (2.43) and colorectal cancers (1.83) in relatives. The SIR was 1.63 for CUP with metastases in the abdomen when a relative was diagnosed with ovarian cancer. CUP with liver metastases associated with liver (1.44) cancer in relatives. CUP with head and neck region metastases associated with relatives’ esophageal (2.87) cancer. CUP metastases in the thorax associated with a relative’s cancers in the upper aerodigestive tract (2.14) and lung (1.74). The findings, matching metastatic location in CUP and primary cancer in relatives, could be reconciled if these cases of CUP constitute a phenotypically modified primary lacking tissue identification, resulting from epitope immunoediting. Alternatively, CUP metastases arise in a genetically favored tissue environment (soil) promoting growth of both primary cancers and metastases (seeds).

In cancer of unknown primary (CUP) the primary site remains unidentified and it has been suggested to be a syndrome with specific clinical characteristics featuring aggressive growth^1^,^2^,^3^. Recommended diagnostic approaches to identify the primary site include detailed histopathological examination with specific immunohistochemistry (IHC) and comprehensive radiological assessment with computed tomography scan of the thorax, abdomen, and pelvis^4^. Improved diagnostic tools and evolving molecular methods of tissue-of-origin identification increase the likelihood of finding the hidden primary tumor, probably contributing to a decrease in CUP incidence^5^,^6^. Although it can be proposed that the frequency of CUP diagnosis is impacted by the breadth and depth of the diagnostic arsenal employed, there seems to be a subgroup of tumor metastases which lack any clues with regard to tissue of origin even subsequent to profound investigation^5^,^6^,^7^. In the Nordic countries, CUP incidence increased until 1995–2000, followed by a sharp continued decline^8^,^9^. In the USA the decline started already in around 1980^10^. These patterns are compatible with an initial increase in the capacity for sensitive diagnosis of tumor metastases (by computer tomography) and a subsequent increase in the ability of pathologists to specifically define tissue of origin (by immunohistochemistry)^7^,^11^. In spite of the declining incidence, CUP accounts for 2–4% of all cancers^8^,^9^,^10^. CUP has a dismal prognosis and it thus ranks the third or fourth among cancer deaths^10^,^12^. In Sweden, the median survival for CUP overall and for the common histologies, adenocarcinoma and undifferentiated carcinoma, was 3 months^13^,^14^. Even though the diagnostics of CUP have improved over time the survival has remained largely unchanged and worst for all cancers over many decades, suggesting that the disease entity has remained uniform^9^,^14^.

The natural history and pathobiology of CUP are poorly understood, chiefly because of the elusive nature of the primary tumor, which precludes evidence-based treatment, compounded by rapid metastatic spread to multiple organs, a feature compatible with escape variants lacking any immunological control^1^,^3^,^15^,^16^. Epithelial–mesenchymal transition (EMT) is believed to be a pivotal process in the metastatic cascade enabling epithelial cell to irreversibly lose cell to cell contacts and acquiring mesenchymal characteristics^17^,^18^. Another process which may destroy cellular features recognized by the IHC arsenal used in tissue identification could be immunoediting^19^,^20^. The immune system is capable of eradicating tumor clones and editing out immunogenic features. The edited clones are therefore poorly immunogenic or even immune suppressive (‘stealth clones’), enabling rapid expansion characteristics such as seen in many CUP cases^11^. So far genetic studies on CUP have not revealed germline variants that would predispose to this cancer^16^. However, our previous family study showed strong evidence that CUP is not a random condition but the risk associates with defined primary cancers in family members^2^. Thus CUP was found in excess in patients whose family members were diagnosed with either CUP or with lung, liver, kidney and other cancers. The cause of death in CUP patients was frequently assigned to the fatal organ metastasis, and, curiously, CUP patients died often from metastases in the lungs, liver, kidneys or other organs when their relatives were diagnosed with primary cancers at the same sites^11^. We speculated that the results could imply shared genetic mechanisms between certain primary cancers and CUP. The rapidly increasing understanding of the interplay of the immune system and carcinogenesis supports the alternative or complementary hypothesis that CUP in a certain organ may in fact be a an immunologically edited version of a tumor arising from the same organ. Shared environmental risk factors could be contributing to these findings but smoking is so far the only established risk factor and in fact the magnitude of its effect is much weaker than in lung cancer^21^,^22^.

In the present study we wanted to examine the familial associations of CUP using the recently updated Swedish Family-Cancer Database with 56,049 patients with CUP diagnosis. Special focus was applied to familial risks between primary cancer and CUP with defined metastatic locations. The hypothesis was that if the metastatic location matched the primary site in a family member, CUP could in these cases be a phenotypically (eg. immunologically) modified primary cancer, redefining the “U” of what used to be CUP. In essence we propose that CUP diagnosis reflects some degree of deficiency in the usual tissue typing tools (antibodies) to identify immunological escape variants. This is not illogical, given that the mentioned antibodies probably recognize the very epitopes likely to be immunoedited in escape variants.

## Results

In total, 5506 offspring with CUP had a first-degree relative with some cancer with an overall SIR of 1.05 (Table 1). As a total of 9171 offspring were diagnosed with CUP, 60% had a relative with any cancer. Cancer sites were included in the Tables when at least 50 cases were found for ‘First degree relatives’; yet the bottom line ‘All’ includes all cancer sites. The overall SIR was not increased (0.99) when a parent was diagnosed with any cancer, for siblings the SIR was 1.09, and for offspring when a parent and a sibling were affected it was 1.24. For concordant CUP, only the risk between siblings was increased (1.40). Offspring CUP was associated with 2 discordant cancer sites in parents, 6 discordant cancers among siblings and 4 cancers (counting colon and colorectum as one) in parents and siblings. Among siblings, high SIRs were found for CUP and connective tissue tumors (1.91) and CUP and lung cancer (1.71). For multiplex families (parent and offspring affected) CUP associated with liver (3.94), ovarian (3.41), lung (2.43) and colorectal cancers (1.83). None of the multiplex families had more than 3 affected individuals, i.e., offspring with CUP and a sibling and a parent with cancer X; thus no extensive pedigrees were available. The median age of onset of CUP in the offspring generation was 59 years and it was unchanged in familial pairs of significant risk in Table 1.

As the incidence of CUP has changed over time it would be relevant to consider possible periodic changes in familial risk. However, a proper analysis is difficult because parents and offspring belong to different generations with different background rates for CUP and different age constrains (cf. Methods). Offspring risk of CUP (parent diagnosed with CUP) was practically unchanged whether the diagnosis was before year 2000 (1.06) or later (1.08, both SIRs non-significant).

The analyses in reverse order, i.e., risk of cancer in offspring when a relative was diagnosed with CUP are shown in Table 2. The SIRs were increased for 12 cancers when parents were diagnosed with CUP. The risks were highest for ovarian (1.30), liver (1.28) and colon cancers (1.26). The risks between siblings were not completely independent from Table 1 because the affected sibling pairs were identical but person-years at risk differed. New associations compared to Table 1, colorectal (and colon), pancreatic, prostate and kidney cancers associated with CUP. The SIR was 2.36 for lung cancer when a parent and a sibling were diagnosed with CUP. There were no deviations in ages of onset in familial cases compared non-familial cases.

In Table 3 risks of CUP in offspring with extranodal metastases are shown depending on the location of metastases and cancers in first degree relatives. For all extranodal metastases, concordant CUP and 6 discordant cancers associated. For simplicity, only sites with some significant associations were shown. When CUP metastases were found in the abdomen, the SIR was 1.63 when a relative was diagnosed with ovarian cancer and it was 1.29 when a relative was diagnosed with stomach cancer. CUP with liver metastases associated with liver (1.44), lung (1.41) and breast (1.19) cancers in relatives. For CUP with head and neck metastases the only associations were to relatives’ esophageal (2.87) and lung (1.77) cancers. Metastases in the thorax associated with a relative’s CUP (1.93) and cancers in the upper aerodigestive tract (2.14) and lung (1.74).

Cancer risks in offspring are shown by location of CUP metastases in relatives in Table 4. Note that in order to make the results completely independent from those in Table 3, only parental CUP was considered. When relatives had any extranodal metastases, 7 cancers were increased in offspring, including kidney and nervous system cancers as novel sites. Ovarian cancer was increased to 1.77 or 1.64 when a parent was diagnosed with abdominal or liver CUP, respectively. Prostate and colon cancer associated also with parental liver cancer. Upper aerodigestive tract cancer was increased to 2.56 and kidney cancer to 2.90 when a parent was diagnosed with head and neck metastases. Endometrial (1.89) and prostate (1.51) cancers were in excess when parents presented with thorax metastases.

CUP is diagnosed in various histological types (cf. Methods) and we tested whether there might be histological concordance in 2 family members, one presenting with CUP and the other with lung cancer, or one with CUP of melanoma histology and the other with melanoma (Table 5). For offspring CUP of squamous cell histology the SIR was 1.29 when a parent was diagnosed with lung cancer of the same histology. For adenocarcinoma histology the SIR for CUP was 1.67 by sibling lung adenocarcinoma. For CUP with melanoma histology the SIRs were 2.15 and 2.14 when a parent of a sibling were diagnosed with melanoma.

## Discussion

We found an intriguing clustering of CUP with many primary cancers in the context of defined families^2^. We found not only familial clustering but also association of metastatic location with the affected organ system in the family member. Salient examples were high risk of offspring abdominal metastatic CUP when relatives were diagnosed with ovarian and stomach cancers, CUP with liver metastases when relatives were diagnosed with liver cancer, CUP with head and neck region metastases when relatives were diagnosed with esophageal cancer and CUP with thorax metastases when relatives were diagnosed with lung cancer. Ovarian cancer association was strong also in the reverse analysis, i.e., ovarian cancer risk in offspring by parental metastatic location in the abdomen. Even stronger was the association of offspring upper aerodigestive tract cancer with parental head and neck metastases. Some additional associations were logical when considering well appreciated metastatic patterns, for example, offspring CUP liver metastases relating to relatives’ lung and breast cancers or offspring liver CUP metastases relating to parental prostate and colon cancers^23^,^24^. Some significant associations initially seemed less obvious. For example, offspring thorax metastases associated with parental endometrial cancer. However, in a large autopsy series, the lung was the most common extranodal metastatic site from uterine cancer^23^.

The possible concordance in histology is another interesting question. However, based on the cancer registry data most cancers are adenocarcinomas without further specification. Lung cancer is an exception as several histological types are recorded and the case numbers allow a detailed analysis. We showed here that there was concordance for histology between family members diagnosed with CUP and lung cancer. The risk of CUP was 1.11 (not significant) in offspring whose parents were diagnosed with melanoma (Table 2) but the risk was increased to 2.15 when CUP of rare melanoma histology was considered (Table 5).

The strength of the associations of site specific CUP metastasis and matching site specific primary cancer reached up to the level of familial associations between concordant primary cancers which usually show SIRs of around 2.0^25^. This is remarkable because of the incidence changes in CUP over the several decades covered in the study and most likely affecting parent-offspring comparisons where the periodic difference in diagnoses of the two generations may span decades. Furthermore, there may some inconsistencies in diagnosis and reporting of metastatic sites in CUP, particularly in moribund patients. The present kind of ‘agnostic’ studies typically apply multiple comparisons whereby some associations are likely to be false positives. Thus the consistency of the two reversed ways of analysis increases credibility of the findings. But how can we explain these results?

One possible explanation to the findings could be shared risk factors but so far only smoking has been consistently associated with the risk of CUP, and only mainly with CUP with lung metastases^21^,^22^. We know from previous experience that concordant cancers have a familial risk of about 2.0 between first-degree relatives while discordant associations, if any, tend to reach SIRs no higher than 1.1 and 1.2, implying the genes underlying familial cancer have site-specific manifestations^26^. Thus there would be good reasons to assume that some genetic factors result in shared predisposition or a fertile ‘soil’ to both a defined primary and CUP metastasis, representing the ‘seed’^27^.

The simplest explanation to the findings would be that in fact the ‘hidden primary’ resides in the organ site marked by familial cancer, and due to immunoediting or other types of phenotypic plasticity it cannot be recognized as originating from that organ^28^. Some support to this hypothesis comes from the implicated primary cancers: lung, ovarian, liver and colorectal cancers which do metastasize to distant organs but are also characterized by extensive local growth with fatal consequences^29^,^30^,^31^. One could even propose that CUP could stand for “Cancer Undefinable by modern immunoPathology’’ (because of lack of tissue determining epitopes), in addition to the conventional definition. Of course, this is about to change because of the increasing diagnostic application of non-IHC based tissue-of-origin methods relying on molecular genetic and gene expression signatures^32^,^33^,^34^. A second theory could invoke the soil part of the common ‘seed and soil’ paradigm^27^. Accordingly, the genetic constitution fosters a favorable environment both to cancer initiation (relative’s primary cancer) and to metastatic seeding (offspring CUP). The common denominator to these theories could be genetic predisposition to faltering immune surveillance which would be permissive to both the growth of primary tumors and seeding of metastases.

In Table 1 we saw that CUP risk is quite high in ‘multiplex’ families in which a parent and a sibling were diagnosed with the same cancer. E.g. CUP risk was 2.43 when the two other family members were diagnosed with lung cancer. Interestingly, this is equally high as the risk for lung cancer when two family members were diagnosed with lung cancer (SIR 2.46, Frank *et al.* unpublished). These data suggest that CUP is reinforcing familial risk as if it were a concordant cancer, with implications about the phenotypic modification of the original cell type. For colorectal (SIR 1.83 in multiplex families), liver (3.94), lung (2.43) and ovarian (3.41) cancers CUP may imply a rare high-risk phenotype and may offer a signal to the oncologist taking a family history. It was also remarkable that a high percentage of 60% of offspring with CUP had a family member with some cancer.

The relative risks reported in this paper are approximately in the range of concordant primary cancers, such as breast, prostate, lung and colorectal cancers^35^. However, relative risk is not a tangible measure for an individual and thus clinical genetic counseling prefers absolute risks which consider disease prevalence. In Sweden the cumulative incidence of CUP by age 75 is 0.75%, slightly higher than those for pancreatic or kidney cancers^36^. Thus a SIR of 1.45 for a sibling of a CUP patient would translate to an absolute risk of 1.1%, or an extra risk of 0.35% units. The cumulative risk by age 75 for all cancer is 29.5% in Sweden^36^.

In summary, although CUP has been considered a heterogeneous syndrome it shows remarkable non-randomness with regard to familial risks of many primary cancers. CUP is a component cancer in relatively high-risk clusters involving colorectal, liver, lung and ovarian cancers. We speculate that the findings related to familial clustering could be reconciled by two non-mutually exclusive phenomena: a) many cases of CUP may constitute a phenotypically modified primary cancer which cannot be identified as such because modern pathology relies heavily on IHC, which in turn depends on tissue epitopes being present. If absent, or immunoedited out, pathological diagnosis could be compromised. b) CUP metastasis may arise in a genetically favored tissue promoting growth of both primary cancers and metastases as predicted by the seed and soil paradigm. Importantly, scenarios a) and b) do not exclude each other and thus both of them could play a role in many cases of CUP.

## Patients and Methods

Cancer cases were retrieved from the Swedish Cancer Registry and combined individually with population databases at Center for Primary Health Care Research, Malmö, Lund University. These Swedish registers, provided by Statistics Sweden, included national census data (1960–1990) with information on individual’s socioeconomic status, the Swedish population register (1990–2012) and the Multigeneration Register, containing the population in families, and constituting the Swedish Family-Cancer Database, used by us in numerous studies^37^. In the Database, the offspring generation was born after 1931 and the parental population was born any time earlier. In the current Database the offspring generation has reached age 80 years; siblings can be defined only in the offspring generation. All linkages were performed using the national ten-digit civic identification number that is assigned to each person in Sweden for his or her lifetime. This number was replaced by a serial number for each person in order to provide anonymity.

The Cancer Registry uses International Classification of Diseases (ICD) codes to identify malignant tumors. CUP was identified with ICD-7 code 199. In total we had 56,049 CUP patients, of which 26,689 (47.6%) had adenocarcinoma, 9444 (16.8%) undifferentiated carcinoma, 1801 (3.2%) melanoma and 2538 (4.5%) with squamous cell cancer; the rest had miscellaneous or missing histology. ICD-9 codes, available from 1987 onwards, were used to identify the anatomic site where metastases were found. These included ‘unspecified CUP’ (ICD-9 code 1990–1991, metastases often spread to multiple organs), ‘liver CUP’ with liver metastasis, ‘thorax CUP’ with lung involvement (including thorax and pleura), ‘abdominal CUP’ with abdominal metastases (including ovary) and ‘other CUP’ with other metastatic locations (any other specified site). A total of 33,677 patients were diagnosed with an ICD-9 code, including 7210 (21.4%) with metastases located in the thorax, 6064 (18.0%) located in the liver, 6414 (19.0%) located in the abdomen and 11,433 (33.9%) located in undefined and multiple sites; the remaining 8% were rare metastasis in the bone, brain and skin.

Family relationships were defined by mutually exclusive probands: parent only, sibling only, parent and sibling, and any first degree relative. Cancer risks were calculated for offspring CUP by cancer in a proband, or in reverse order, for offspring cancer by CUP in a proband. Note that for parental probands the two ways of calculation are completely independent. Person-years were calculated from the offspring date of birth, depending on the family history, until diagnosis of cancer, death, emigration or closing date (December 31, 2012), whichever came first. Only the first diagnosed cancer was considered. Standardized incidence ratios (SIRs) were calculated as the ratio of observed to expected number of cases^38^. The expected numbers were calculated for all individuals without a history of a specific cancer, and the rates were standardized by 5-year-age, gender, period (5 years group), socioeconomic status and residential area^39^. The 95% confidence interval (95%CI) of the SIR was calculated assuming a Poisson distribution, and they were rounded to the nearest two decimals^39^. Associations are called only when 95%CIs did not include 1.00. All analyses were performed using the SAS statistical package (version 9.1; SAS Institute, Cary, NC).

### Ethical statement

The study was approved by the Ethical Committee of Lund University and the study was conducted in accordance with the approved guidelines.

## Additional Information

**How to cite this article**: Hemminki, K. *et al.* Location of metastases in cancer of unknown primary are not random and signal familial clustering. *Sci. Rep.* **6**, 22891; doi: 10.1038/srep22891 (2016).

## Figures and Tables

**Table 1:** Risk of cancer of unknown primary for offspring with first degree relatives diagnosed with cancer.

Cancer in relatives	Parental only				Sibling only				Parent and sibling				First degree relatives			
	O	SIR	95%CI		O	SIR	95%CI		O	SIR	95%CI		O	SIR	95%CI	
Upper aerodigestive tract	146	**1.33**	1.12	1.56	58	1.12	0.85	1.44	2	1.94	0.18	7.13	206	**1.26**	1.10	1.45
Esophagus	52	1.28	0.95	1.68	23	1.28	0.81	1.92	1	7.22	0.00	41.39	76	**1.29**	1.02	1.62
Stomach	239	0.96	0.84	1.09	44	1.13	0.82	1.51	3	1.31	0.25	3.87	286	0.98	0.87	1.10
Colorectum	564	0.96	0.88	1.04	237	1.12	0.98	1.26	24	1.83	1.17	2.63	825	1.01	0.95	1.08
Colon	347	0.92	0.83	1.02	134	1.03	0.87	1.23	18	**1.78**	1.05	2.81	499	0.97	0.88	1.05
Rectum	217	1.02	0.89	1.16	103	**1.25**	1.02	1.51	6	1.98	0.71	4.35	326	1.09	0.98	1.22
Liver	177	1.14	0.98	1.33	54	**1.40**	1.05	1.83	4	**3.94**	1.03	10.19	235	**1.21**	1.06	1.38
Pancreas	152	1.01	0.85	1.18	54	1.19	0.90	1.56	4	2.87	0.75	7.42	210	1.06	0.92	1.22
Lung	404	**1.19**	1.07	1.31	284	**1.71**	1.52	1.92	25	**2.43**	1.57	3.59	713	**1.38**	1.28	1.48
Breast	484	1.00	0.91	1.10	435	**1.17**	1.06	1.29	35	1.10	0.77	1.53	954	**1.08**	1.01	1.15
Cervix	103	1.09	0.89	1.33	44	1.09	0.79	1.46	0				147	1.09	0.92	1.28
Endometrium	125	0.96	0.80	1.15	68	0.99	0.77	1.26	2	1.10	0.10	4.04	195	0.97	0.84	1.12
Ovary	121	1.12	0.93	1.34	62	1.07	0.82	1.37	5	**3.41**	1.08	8.03	188	1.12	0.97	1.30
Prostate	706	1.05	0.98	1.13	361	1.07	0.96	1.19	48	0.91	0.67	1.21	1115	1.05	0.99	1.12
Kidney	171	1.11	0.95	1.29	69	1.22	0.95	1.54	3	1.96	0.37	5.81	243	**1.14**	1.00	1.30
Urinary bladder	264	1.12	0.99	1.26	104	1.14	0.93	1.39	3	0.75	0.14	2.21	371	**1.12**	1.01	1.24
Melanoma	114	1.11	0.92	1.33	126	1.08	0.90	1.28	4	1.43	0.37	3.70	244	1.10	0.96	1.24
Skin, squamous cell	209	0.96	0.83	1.10	69	1.04	0.81	1.32	3	0.91	0.17	2.68	281	0.98	0.87	1.10
Nervous system	127	1.08	0.90	1.29	95	1.03	0.83	1.26	1	0.52	0.00	3.01	223	1.05	0.92	1.20
Thyroid gland	42	1.24	0.90	1.68	24	1.02	0.65	1.52	0				66	1.15	0.89	1.46
Endocrine glands	76	1.06	0.83	1.32	54	1.10	0.83	1.44	1	1.36	0.00	7.80	131	1.08	0.90	1.28
Connective tissue	29	0.97	0.65	1.39	31	**1.91**	1.30	2.71	1	11.33	0.00	64.92	61	**1.32**	1.01	1.69
Non-Hodgkin lymphoma	185	1.00	0.86	1.16	104	1.05	0.86	1.28	2	0.54	0.05	1.98	291	1.01	0.90	1.14
Myeloma	85	1.10	0.88	1.36	25	0.90	0.58	1.33	1	1.62	0.00	9.27	111	1.05	0.86	1.27
Leukemia	95	1.08	0.87	1.32	44	1.04	0.76	1.40	0				139	1.06	0.89	1.26
CUP	189	1.07	0.93	1.24	85	**1.40**	1.12	1.73	0				274	**1.15**	1.02	1.30
All	3188	0.99	0.96	1.03	1133	**1.09**	1.02	1.15	172	**1.24**	1.06	1.43	5506	**1.05**	1.02	1.08

O = observed number of cases in offspring. Bolding shows that the 95% confidence limits (95%CI) do not include 1.00.

**Table 2:** Risk of cancer in offspring with first degree relatives diagnosed with CUP.

Cancer in offspring	Parental only				Sibling only				Parent and sibling				First degree relatives			
	O	SIR	95%CI		O	SIR	95%CI		O	SIR	95%CI		O	SIR	95%CI	
Upper aerodigestive tract	188	**1.18**	1.02	1.36	59	1.15	0.88	1.49	1	0.95	0.00	5.45	248	**1.17**	1.03	1.33
Esophagus	61	1.15	0.88	1.47	25	1.33	0.86	1.96	0				86	1.19	0.95	1.47
Stomach	97	0.97	0.79	1.19	46	1.33	0.97	1.78	0				143	1.06	0.89	1.25
Colorectum	754	1.23	1.14	1.32	270	**1.27**	1.12	1.43	7	1.60	0.63	3.00	1031	1.24	1.17	1.32
Colon	479	**1.26**	1.15	1.38	162	**1.23**	1.05	1.44	4	1.47	0.38	3.80	645	**1.25**	1.16	1.36
Rectum	275	**1.18**	1.05	1.33	108	**1.34**	1.10	1.62	3	1.80	0.34	5.32	386	**1.23**	1.11	1.35
Liver	136	**1.28**	1.08	1.52	55	**1.48**	1.11	1.93	1	1.31	0.00	7.51	192	**1.33**	1.15	1.54
Pancreas	151	**1.25**	1.06	1.47	62	**1.45**	1.11	1.86	0				213	**1.30**	1.13	1.48
Lung	542	**1.22**	1.12	1.33	305	**1.91**	1.70	2.13	8	**2.36**	1.01	4.67	855	**1.41**	1.32	1.51
Breast	1487	**1.11**	1.06	1.17	505	**1.24**	1.13	1.35	9	1.01	0.46	1.92	2001	**1.14**	1.09	1.19
Cervix	160	1.08	0.92	1.26	47	1.19	0.88	1.59	0				207	1.10	0.95	1.26
Endometrium	226	1.07	0.94	1.22	69	0.90	0.70	1.14	3	1.81	0.34	5.37	298	1.03	0.92	1.15
Ovary	227	**1.30**	1.13	1.48	69	1.22	0.95	1.55	0				296	**1.27**	1.13	1.43
Prostate	1270	**1.19**	1.12	1.25	456	**1.21**	1.11	1.33	7	0.89	0.35	1.83	1733	**1.19**	1.14	1.25
Kidney	186	1.14	0.99	1.32	72	**1.31**	1.02	1.65	0				258	**1.18**	1.04	1.33
Urinary bladder	296	**1.18**	1.05	1.33	101	1.14	0.93	1.39	4	2.21	0.57	5.71	401	**1.18**	1.07	1.30
Melanoma	525	**1.13**	1.04	1.23	133	1.05	0.88	1.24	4	1.49	0.39	3.84	662	**1.12**	1.03	1.20
Skin, squamous cell	211	1.02	0.89	1.17	66	0.94	0.73	1.20	3	2.10	0.40	6.21	280	1.01	0.89	1.13
Nervous system	334	0.99	0.89	1.11	100	1.10	0.90	1.34	1	0.52	0.00	2.99	435	1.01	0.92	1.11
Thyroid gland	104	**1.24**	1.01	1.50	25	1.11	0.72	1.64	0				129	**1.20**	1.00	1.43
Endocrine glands	182	1.09	0.94	1.26	58	1.16	0.88	1.50	1	0.93	0.00	5.32	241	1.11	0.97	1.25
Connective tissue	70	1.26	0.98	1.59	29	**1.87**	1.25	2.69	1	3.14	0.00	17.98	100	**1.40**	1.14	1.70
Non-Hodgkin lymphoma	337	1.11	0.99	1.23	110	1.12	0.92	1.35	0				447	**1.10**	1.00	1.21
Myeloma	90	1.18	0.95	1.45	27	1.04	0.69	1.52	0				117	1.14	0.94	1.37
Leukemia	160	1.05	0.90	1.23	42	1.05	0.76	1.42	0				202	1.05	0.91	1.20
CUP	189	1.12	0.97	1.29	85	**1.45**	1.16	1.79	0				274	**1.20**	1.06	1.35
All	8342	**1.14**	1.12	1.17	2903	**1.24**	1.20	1.29	52	1.05	0.79	1.38	11297	**1.17**	1.15	1.19

O = observed number of cases in offspring. Bolding shows that the 95% confidence limits (95%CI) do not include 1.00.

**Table 3:** Risk of cancer of unknown primary with defined extranodal metastases for offspring with first degree relatives diagnosed with cancer.

Cancer in relatives	All				Abdomen				Liver				Head & neck				Thorax			
	O	SIR	95%CI		O	SIR	95%CI		O	SIR	95%CI		O	SIR	95%CI		O	SIR	95%CI	
Upper aerodigestive tract	171	**1.25**	1.07	1.45	40	1.29	0.92	1.75	33	1.22	0.84	1.71	7	0.91	0.36	1.88	15	**2.14**	1.19	3.54
Esophagus	57	1.15	0.87	1.49	15	1.34	0.75	2.21	9	0.92	0.42	1.75	8	**2.87**	1.22	5.68	4	1.58	0.41	4.08
Stomach	248	1.00	0.88	1.13	72	**1.29**	1.01	1.63	42	0.88	0.63	1.18	15	1.17	0.65	1.94	14	1.16	0.63	1.95
Colorectum	691	1.00	0.93	1.08	164	1.05	0.89	1.21	146	1.09	0.92	1.27	30	0.79	0.53	1.10	34	1.00	0.69	1.36
Colon	418	0.96	0.87	1.06	102	1.03	0.84	1.25	92	1.08	0.87	1.32	14	0.58	0.32	0.98	19	0.88	0.53	1.37
Rectum	273	1.08	0.96	1.22	62	1.08	0.83	1.39	54	1.10	0.83	1.44	16	1.16	0.66	1.89	15	1.20	0.67	1.99
Liver	187	1.14	0.98	1.31	45	1.21	0.88	1.62	46	**1.44**	1.05	1.92	13	1.47	0.78	2.52	6	0.74	0.27	1.62
Lung	603	**1.39**	1.28	1.51	113	1.15	0.95	1.39	121	**1.41**	1.17	1.68	44	**1.77**	1.29	2.38	39	**1.74**	1.24	2.38
Breast	805	**1.08**	1.01	1.16	195	1.15	1.00	1.33	172	**1.19**	1.02	1.38	51	1.23	0.92	1.62	37	1.00	0.70	1.38
Endometrium	166	0.98	0.84	1.15	44	1.15	0.84	1.55	33	1.01	0.70	1.42	10	1.08	0.52	2.00	9	1.11	0.50	2.11
Ovary	167	**1.18**	1.01	1.38	52	**1.63**	1.22	2.14	36	1.31	0.92	1.82	10	1.30	0.62	2.39	3	0.43	0.08	1.27
Prostate	957	**1.07**	1.01	1.14	223	1.10	0.96	1.25	180	1.04	0.90	1.21	57	1.17	0.89	1.52	36	0.82	0.57	1.14
Kidney	206	1.15	1.00	1.31	46	1.13	0.83	1.51	37	1.07	0.75	1.47	14	1.45	0.79	2.44	7	0.80	0.32	1.66
Urinary bladder	317	**1.14**	1.02	1.27	66	1.04	0.81	1.33	54	0.99	0.74	1.29	18	1.15	0.68	1.82	20	1.43	0.87	2.21
Nervous system	186	1.05	0.90	1.21	45	1.12	0.81	1.50	39	1.14	0.81	1.56	9	0.92	0.42	1.76	12	1.38	0.71	2.42
CUP	236	**1.17**	1.03	1.33	53	1.17	0.87	1.53	45	1.16	0.84	1.55	7	0.64	0.25	1.33	19	**1.93**	1.16	3.02
All	4635	**1.05**	1.02	1.08	1062	1.06	1.00	1.12	903	1.05	0.99	1.12	269	1.10	0.98	1.24	227	1.04	0.91	1.18

**Table 4:** Risk of cancer in offspring with parents diagnosed with cancer of unknown primary with defined extranodal metastases.

Cancer	All				Abdomen				Liver				Head & neck				Thorax			
	O	SIR	95%CI		O	SIR	95%CI		O	SIR	95%CI		O	SIR	95%CI		O	SIR	95%CI	
Upper aerodigestive tract	95	1.13	0.91	1.38	20	0.93	0.57	1.44	13	0.72	0.38	1.23	7	**2.56**	1.02	5.31	8	1.10	0.47	2.17
Esophagus	30	1.08	0.73	1.55	5	0.71	0.22	1.66	8	1.36	0.58	2.68	2	2.41	0.23	8.85	3	1.26	0.24	3.74
Stomach	34	0.67	0.46	0.94	6	0.47	0.17	1.02	8	0.76	0.32	1.50	0				2	0.49	0.05	1.80
Colorectum	375	1.24	1.12	1.37	90	1.16	0.94	1.42	82	1.30	1.03	1.60	10	1.10	0.53	1.89	34	1.37	0.95	1.87
Colon	236	**1.28**	1.12	1.45	59	1.25	0.95	1.62	52	**1.35**	1.01	1.77	8	1.44	0.61	2.85	20	1.33	0.81	2.05
Rectum	139	1.18	0.99	1.39	31	1.03	0.70	1.47	30	1.22	0.82	1.74	2	0.57	0.05	2.08	14	1.44	0.78	2.42
Liver	58	1.08	0.82	1.40	10	0.73	0.35	1.35	15	1.33	0.74	2.21	3	1.84	0.35	5.44	7	1.56	0.62	3.23
Lung	287	**1.28**	1.13	1.43	80	**1.39**	1.10	1.73	52	1.10	0.82	1.44	10	1.51	0.72	2.80	21	1.08	0.67	1.65
Breast	786	**1.08**	1.01	1.16	207	1.12	0.97	1.28	165	1.08	0.92	1.26	23	1.00	0.63	1.50	63	1.06	0.81	1.36
Endometrium	120	1.15	0.96	1.38	21	0.79	0.49	1.21	21	0.98	0.61	1.50	6	2.11	0.76	4.63	16	**1.89**	1.08	3.08
Ovary	106	**1.24**	1.02	1.51	38	**1.77**	1.25	2.43	29	**1.64**	1.10	2.36	2	0.77	0.07	2.84	10	1.47	0.70	2.71
Prostate	636	**1.20**	1.11	1.30	162	**1.18**	1.01	1.38	138	**1.24**	1.04	1.47	22	1.43	0.89	2.16	69	**1.51**	1.18	1.91
Kidney	101	**1.27**	1.03	1.54	25	1.24	0.80	1.83	18	1.08	0.64	1.71	7	**2.90**	1.15	6.02	8	1.24	0.53	2.45
Urinary bladder	130	1.08	0.90	1.28	31	1.01	0.68	1.43	27	1.06	0.70	1.55	5	1.39	0.44	3.27	11	1.08	0.53	1.93
Nervous system	192	**1.16**	1.00	1.33	51	1.24	0.92	1.63	46	1.33	0.97	1.77	7	1.21	0.48	2.52	12	0.95	0.49	1.66
CUP	92	1.06	0.86	1.31	15	0.68	0.38	1.13	19	1.05	0.63	1.64	5	1.94	0.61	4.57	10	1.41	0.67	2.61
All	4210	**1.14**	1.11	1.18	1044	**1.12**	1.05	1.19	873	**1.13**	1.06	1.21	153	**1.31**	1.11	1.54	368	**1.22**	1.10	1.35

**Table 5:** Risk of cancer of unknown primary of specific histology when a parent or a sibling was diagnosed with lung cancer or melanoma with a matching histology.

	Parental history				Sibling history			
Lung squamous cell	73	**1.29**	1.01	1.63	4	1.90	0.49	4.92
Lung adenocarcinoma	41	1.12	0.81	1.53	62	**1.67**	1.28	2.15
Melanoma	21	**2.15**	1.33	3.29	19	**2.14**	1.29	3.35
